# Supplementary material for: Theta oscillations optimize a speed-precision trade-off in phase coding neurons
Source: PLoS Comput Biol. 2024 Dec 2;20(12):e1012628. doi: 10.1371/journal.pcbi.1012628 (PMC11637358; doi:10.1371/journal.pcbi.1012628)
Supplement: S1 Appendix — Provides a detailed solution to the deterministic part of Eq 1, resulting in the “rate-to-phase” transfer function (Eq 3) previously derived in [6]. (PDF) [file pcbi.1012628.s001.pdf]

S1 Appendix. Full derivation of the mean phase of firing

To determine the expected spike phase, we focus on the deterministic part of the neuron model, assuming small noise amplitudes and Gaussian distributions. The general solution for an equation of the form:

$$\tau_m \frac{dV}{dt} = -V + R_m I(t) \tag{1}$$

is given by:

$$V(t) = V_0 e^{-(t-t_0)/\tau_m} + \frac{R_m}{\tau_m} \int_{t_0}^{t} e^{-(t-t')/\tau_m} I(t') dt'. \tag{2}$$

For the current $I(t') = I_s - I_{osc} \cos(\omega t' + \phi)$ in Equation 1 (main text), we compute two integrals. The first integral is:

$$\frac{R_m}{\tau_m} \int_{t_0}^{t} e^{-(t-t')/\tau_m} I_s dt' = R_m I_s \Big(1 - e^{-(t-t_0)/\tau_m}\Big). \tag{3}$$

To handle the sinusoidal component, we use the fact that $\mathrm{Re}[\int f(x)dx] = \int \mathrm{Re}[f(x)]dx$:

$$\frac{R_m}{\tau_m} \int_{t_0}^{t} e^{-(t-t')/\tau_m} I_{osc} \cos(\omega t' + \phi) dt' = \mathrm{Re}\left[\frac{R_m e^{i\phi}}{\tau_m} \int_{t_0}^{t} e^{-(t-t')/\tau_m} I_{osc} e^{i\omega t'} dt'\right] \tag{4}$$

$$= \mathrm{Re}\left[\frac{R_m e^{i\phi}}{\tau_m} \int_{t_0}^{t} e^{-(t-t')/\tau_m} e^{i\omega t'} dt'\right] \tag{5}$$

$$= \mathrm{Re}\left[\frac{R_m e^{i\phi}}{\tau_m} \frac{e^{i\omega t} - e^{i\omega t_0} e^{-(t-t_0)/\tau_m}}{1 + i\omega\tau_m}\right]. \tag{6}$$

We factor out and express in polar coordinates:

$$\frac{e^{i\phi}}{1 + i\omega\tau_m} = e^{i\phi} \frac{1 - i\omega\tau_m}{1 + (\omega\tau_m)^2} = A e^{i\varphi}, \tag{7}$$

where

$$A = \frac{1}{\sqrt{1 + (\tau_m\omega)^2}} \quad , \quad \varphi = -\arctan(\omega\tau_m) + \phi. \tag{8}$$

The real part of the expression is:

$$\mathrm{Re}\left[\frac{R_m e^{i\phi}}{\tau_m} \left(e^{i\omega t} - e^{i\omega t_0} e^{-(t-t_0)/\tau_m}\right)\right] \tag{9}$$

$$= R_m I_{osc} A \left(\cos(\omega t + \varphi) - e^{-(t-t_0)/\tau_m} \cos(\omega t_0 + \varphi)\right). \tag{10}$$

Substituting Equations 3 and 10 into Equation 2, we obtain the membrane potential:

$$\begin{aligned} \mu_V \equiv V(t) = V_0 e^{-(t-t_0)/\tau_m} &+ R_m I_s \left(1 - e^{-(t-t_0)/\tau_m}\right) \\ &- R_m I_{osc} A \left(\cos(\omega t + \varphi) - e^{-(t-t_0)/\tau_m} \cos(\omega t_0 + \varphi)\right). \end{aligned} \tag{11}$$

Then, assuming $V_0 = 0$, the voltage trajectory, given a spike at time t' and initial phase ϕ_0, can be found as:

$$\begin{aligned} V(t) = R_m I_s &\left(1 - e^{-(t-t')/\tau_m}\right) \\ &- R_m I_{osc} A \left(\cos\left(\omega t + \varphi + \phi_0\right) - e^{-(t-t')/\tau_m} \cos\left(\omega t' + \varphi + \phi_0\right)\right). \end{aligned} \tag{12}$$

The spike phase of a neuron in cycle i is defined as the modulo 2π of the time of the first spike within the cycle ($t_i^{(0)}$), multiplied by the angular frequency ω:

$$\phi_i = \left[\omega t_i^{(0)} \mod 2\pi\right] + \phi_0. \tag{13}$$

Imposing $t - t' = t_i^{(0)} - t_{i-1}^{(0)} = T \Longrightarrow t = t_{i-1}^{(0)} + T$, and substituting $\omega t_i^{(0)} = \phi_i - \phi_0$ in Equation 12, we obtain:

$$V_{th} = \left(R_m I_s - R_m I_{osc} A \cos\left(\phi_i + \varphi\right)\right)\left(1 - e^{-T/\tau_m}\right). \tag{14}$$

Isolating ϕ_i, we get:

$$\mu_\phi \equiv \phi_i = \arccos\left(\frac{R_m I_s \left(1 - e^{-T/\tau_m}\right) - V_{th}}{R_m I_{osc} A \left(1 - e^{-T/\tau_m}\right)}\right) - \varphi, \tag{15}$$

which gives the expected phase μ_ϕ at which the neurons will lock given an I_s value, replicating the main result in [1]. Importantly, this equation constraints the range of I_s where the neuron will be within the phase-locking regime. To determine such a range, one must find the domain of the function, i.e., where $-1 \leq \arccos \leq 1$ holds. Solving these inequalities, we obtain:

$$\min(I_s) = \left(\frac{V_{th}}{R_m I_{osc} A \left(1 - e^{-T/\tau_m}\right)} - 1\right) A I_{osc}, \tag{16}$$

$$\max(I_s) = \left(\frac{V_{th}}{R_m I_{osc} A \left(1 - e^{-T/\tau_m}\right)} + 1\right) A I_{osc}. \tag{17}$$

For simulations, we select M equally-spaced values between minimum and maximum, leaving a fraction (20%) of unused range on both extremes to alleviate boundary effects.

References

1. McLelland D, Paulsen O. Neuronal oscillations and the rate-to-phase transform: mechanism, model and mutual information. The Journal of physiology. 2009;587(4):769–785.
