# Supplementary material for: Theta oscillations optimize a speed-precision trade-off in phase coding neurons
Source: PLoS Comput Biol. 2024 Dec 2;20(12):e1012628. doi: 10.1371/journal.pcbi.1012628 (PMC11637358; doi:10.1371/journal.pcbi.1012628)
Supplement: S2 Appendix — Explains the use of a first-order Taylor series expansion and the propagation of uncertainty around the spike threshold to derive an analytical approximation of the phase variance. (PDF) [file pcbi.1012628.s002.pdf]

S2 Appendix. Full derivation of the variance of phase of firing

To estimate the phase variance (σ_ϕ^2) due to neuronal noise, we consider the variance in spike timing (σ_t^2) induced by fluctuations in the membrane potential (see Fig 3A in main text). We employ a first-order Taylor series expansion and the propagation of uncertainty to derive an analytical approximation, along the lines of previous work [1, 2].

The membrane potential $V(t)$ is approximated linearly around the spike threshold V_{th}. We represent the membrane potential as $V(t) = V_{th} + \delta V$, where δV is a small deviation from the threshold. Using a Taylor series expansion, the potential at a slightly later time $t + \delta t$ is given by:

$$V(t + \delta t) \approx V(t) + \frac{dV}{dt}\delta t. \tag{1}$$

Since $V(t + \delta t) - V(t) = \delta V$, by rearranging the terms, we find the deviation in time as a function of the deviation in potential:

$$\delta t \approx \frac{\delta V}{\frac{dV}{dt}}. \tag{2}$$

The variance in spike timing, σ_t^2, is related to the fluctuations in membrane potential (δV). Using the propagation of uncertainty, we express σ_t^2 as:

$$\sigma_t^2 \approx \left(\frac{\partial \delta t}{\partial \delta V}\right)^2 \sigma_{\delta V}^2. \tag{3}$$

Substituting $\frac{\partial \delta t}{\partial \delta V} = \frac{1}{\frac{dV}{dt}}$ and recognizing that $\sigma_{\delta V}^2$ is the variance in membrane potential ($\sigma_V^2(t)$), which is given by:

$$\sigma_V^2(t) = \mathrm{Var}\left[\int_{t_0}^{t} \sigma_W \xi(t') e^{-(t-t')/\tau_m} dt'\right] = \frac{K^2 I_s^2 \eta^2 V_{th}^2}{2}\left(1 - e^{-2t/\tau_m}\right), \tag{4}$$

we obtain:

$$\sigma_t^2 \approx \frac{\sigma_V^2(t)}{\left(\frac{dV}{dt}\right)^2}. \tag{5}$$

Then, we evaluate the expression at the threshold, so that:

$$\sigma_{t_f}^2 \approx \left.\frac{\sigma_V^2}{\left(\frac{dV}{dt}\right)^2}\right|_{V_{th}}. \tag{6}$$

This approximation is valid under conditions where deviations from the spike threshold are small and the response of the membrane potential near the threshold is approximately linear. Finally, since time and phase are related by $\phi = \omega t$, we have that:

$$\sigma_\phi^2 \approx \omega^2 \sigma_{t_f}^2, \tag{7}$$

and under the assumption that the time of firing t_f will be the expected in the phase-locking regime $\mathbb{E}\left[t_f\right] = T$, we arrive at the expression:

$$\sigma_\phi^2 \approx \frac{\omega^2 K^2 I_s^2 \eta^2 V_{th}^2 (1 - e^{-2T/\tau_m})\tau_m^2}{2\left(-V_{th} + R_m I_s - R_m I_{osc}\cos\left(\phi_{I_s}\right)\right)^2}. \tag{8}$$

Equation 8 captures how the variance in the phase-of-firing is affected by the accumulation of random fluctuations in the membrane potential $\sigma_V^2(t = T)$, and the membrane potential dynamics around the threshold $\left(\frac{dV}{dt}\right)^2\Big|_{V_{th}}$. Then, since we are assuming small noise amplitudes in the suprathreshold regime (i.e., phase-locking regime) and given that we use linear approximations, the phase probability distribution can be approximated by a Gaussian:

$$p(\phi) = \frac{1}{\sqrt{2\pi\sigma^2}} \exp\left(\frac{[\phi - \mu]^2}{2\sigma^2}\right), \tag{9}$$

with mean $\mu = \phi_{I_s}$, given by Equation 3 (main text), and variance $\sigma^2 = \sigma^2_{\phi_{I_s}}$, given by Equation 4 (main text), for a certain tonic input I_s.

References

1. Kilinc D, Demir A. Spike timing precision of neuronal circuits. Journal of computational neuroscience. 2018;44:341–362.
2. Demir A, Sangiovanni-Vincentelli A. Analysis and simulation of noise in nonlinear electronic circuits and systems. vol. 425. Springer Science & Business Media; 2012.
