# Supplementary material for: Theta oscillations optimize a speed-precision trade-off in phase coding neurons
Source: PLoS Comput Biol. 2024 Dec 2;20(12):e1012628. doi: 10.1371/journal.pcbi.1012628 (PMC11637358; doi:10.1371/journal.pcbi.1012628)
Supplement: S3 Appendix — Describes the approximation of the entropy of Gaussian mixtures to derive an analytical estimation of the information rate. Additionally, it introduces a correction factor to account for cycle-to-cycle correlations. (PDF) [file pcbi.1012628.s003.pdf]

S3 Appendix. Approximation of information rate

We first define the phase as the response R associated with a stimulus S, corresponding to a tonic input current I_s. Assuming M tonic input levels that are equally likely, S is defined as a uniformly distributed variable $p(S = s) = 1/M$. In turn, the unconditioned distribution of R corresponds to a Gaussian mixture with equal weights:

$$p(R = r) = \frac{1}{M} \sum_{m=1}^{M} \frac{1}{\sqrt{2\pi\sigma_m^2}} \exp\left(-\frac{(r - \mu_m)^2}{2\sigma_m^2}\right). \tag{1}$$

Here, μ_m and σ_m^2 are the means and variances associated with each tonic input level I_s (i.e., the Gaussian components). In a Gaussian mixture model, the mutual information $\mathrm{I}(S; R)$ between S and R is given by the difference between the entropy of the Gaussian mixture (unconditioned response) and the average entropy of the individual Gaussian components (response conditioned to the stimulus):

$$\mathrm{I}(S; R) = \mathrm{H}(R) - \frac{1}{M} \sum_{s \in S} \mathrm{H}(R|s). \tag{2}$$

The entropy of a Gaussian mixture $\mathrm{H}(R)$ does not have a known closed-form solution [1]. However, we have found that $\mathrm{H}(R)$ can be well approximated by considering an aggregate measure of the overall spread of the mixture: combining the variance of means $\boldsymbol{\mu}$ and the mean of variances $\boldsymbol{\sigma}^2$, such that:

$$\mathrm{H}(R) \approx \frac{1}{2} \log_2 \left(2\pi e \left(\mathbb{E}[\boldsymbol{\sigma}^2] + \mathrm{Var}[\boldsymbol{\mu}]\right)\right). \tag{3}$$

Then, the average entropy of the individual Gaussian components, $\mathrm{H}(R|S)$, does have a closed-form solution:

$$\mathrm{H}(R|S) = \frac{1}{2} \mathbb{E}\left[\log_2 \left(2\pi e \boldsymbol{\sigma}^2\right)\right]. \tag{4}$$

Combining these, and simplifying, we obtain:

$$\mathrm{I}(S; R) \approx \frac{1}{2} \left(\log_2 \left(\mathbb{E}[\boldsymbol{\sigma}^2] + \mathrm{Var}[\boldsymbol{\mu}]\right) - \mathbb{E}\left[\log_2(\boldsymbol{\sigma}^2)\right]\right), \tag{5}$$

providing a good approximation of the average information of the phase of the first spike in one cycle about the input signal conveyed by I_s.

One could then estimate the information rate r simply by multiplying the information per cycle I and sampling frequency f, such that $\mathrm{r} \approx \mathrm{I}f$. However, this approach assumes statistical independence between cycles, providing only an upper bound on r. Instead, a more realistic approach would be to consider that the oscillation is sampling from an input signal s that has a characteristic time constant τ_s, following an exponential decay in its autocorrelation function $\rho(\Delta t) = e^{-\Delta t/\tau_s}$. Then, the total correlation between one cycle and all subsequent cycles can be represented as a geometric series:

$$C = \sum_{k=1}^{\infty} \rho(kT) = \sum_{k=1}^{\infty} e^{-kT/\tau_s} = \frac{e^{-T/\tau_s}}{1 - e^{-T/\tau_s}}. \tag{6}$$

The sum C represents the cumulative effect of correlations across all cycles. Thus, we define the effective sampling frequency as $f_{\mathrm{eff}} = \frac{f}{1+C} = f(1 - e^{-T/\tau_s})$, with $1 + C$

correcting the information rate by accounting for redundancy, and keeping $f_{\text{eff}} = 0$ as $C \to \infty$ and $f_{\text{eff}} = f$ as $C \to 0$. After simplifying, we get the expression:

$$\mathrm{r} \approx \mathrm{I} f_{\text{eff}} = \mathrm{I} f \left(1 - e^{-T/\tau_s}\right). \tag{7}$$

This expression ensures that the information rate reflects the impact of temporal correlations in the sampled signal, penalizing for oversampling when $T < \tau_s$.

Finally, to find the optimal frequency for a particular set of neuron parameters and noise level, we define the normalized information rate (r_{norm}) as the ratio of r at a specific frequency (r_f) to the sum of r across all frequencies (F):

$$\mathrm{r}_{norm} = \frac{\mathrm{r}_f}{\max_{f \in F}(\mathrm{r}_f)}. \tag{8}$$

The optimal frequency f^* is then defined as the frequency at which r_{norm} is maximized, indicating the frequency that provides the highest information rate:

$$f^* = \arg\max_{f \in F} \left(\mathrm{r}_{norm}\right). \tag{9}$$

This optimal frequency f^* will be determined by the trade-off between sampling speed and encoding accuracy imposed by noise.

References

1. Huber MF, Bailey T, Durrant-Whyte H, Hanebeck UD. On entropy approximation for Gaussian mixture random vectors. In: 2008 IEEE International Conference on Multisensor Fusion and Integration for Intelligent Systems. IEEE; 2008. p. 181–188.
