# Supplementary material for: Theta oscillations optimize a speed-precision trade-off in phase coding neurons
Source: PLoS Comput Biol. 2024 Dec 2;20(12):e1012628. doi: 10.1371/journal.pcbi.1012628 (PMC11637358; doi:10.1371/journal.pcbi.1012628)
Supplement: S4 Appendix — Includes: Table A. Default parameters for hippocampal neurons; Table B. Neuron parameters along the hippocampal dorsoventral axis; Table C. Neuron parameters for visual and olfactory cells. (PDF) [file pcbi.1012628.s004.pdf]

S4 Appendix. Neuron parameters

By default, and unless it is stated otherwise in the main text, we used the parameters shown in Table A. For the neuron and oscillation parameters, we followed [1] and for the noise strength [2], setting $\sigma_W = 0.0135$ V/$\sqrt{\text{s}}$.

Table A. Default parameters for hippocampal neurons.

Parameter	Description	Value
R_m	Membrane resistance	142 MΩ
V_{th}	Spike threshold	15 mV
τ_m	Membrane time constant	24 ms
I_{osc}	Oscillation amplitude	40 pA
σ_W	Noise amplitude	0.0135 V/$\sqrt{\text{s}}$

For the parameter gradients along the dorsoventral axis, we followed [3] and [4]. For the noise strength, we followed [2], setting $\sigma_W = 0.0135$ V/$\sqrt{\text{s}}$ across models. The neuron parameters can be found in Table B.

Table B. Neuron parameters along the hippocampal dorsoventral axis.

Parameter	Dorsal pole	Ventral pole
R_m	24.8 MΩ	94.4 MΩ
V_{th}	20.22 mV	15.78 mV
τ_m	14.45 ms	33.35 ms
I_{osc}	100 pA	40 pA

We based the parameters for pyramidal cells in the primary visual cortex on [6]. For mitral cells in the olfactory bulb, we followed [5], using an oscillatory current of $I_{osc} = 35.4$ pA to produce realistic membrane potential amplitudes of 3.45 mV in response to a 3.7 Hz input oscillation. The list of parameters is provided in Table C.

Table C. Neuron parameters for visual and olfactory cells.

Parameter	Pyramidal cell (V1)	Mitral cell (OB)
R_m	177 MΩ	100 MΩ
V_{th}	28.3 mV	15 mV
τ_m	18.9 ms	10 ms
I_{osc}	40 pA	35.4 pA

References

1. McLelland D, Paulsen O. Neuronal oscillations and the rate-to-phase transform: mechanism, model and mutual information. The Journal of physiology. 2009;587(4):769–785.

2. Lansky P, Sanda P, He J. The parameters of the stochastic leaky integrate-and-fire neuronal model. Journal of Computational Neuroscience. 2006;21(2):211–223.

3. Malik R, Dougherty KA, Parikh K, Byrne C, Johnston D. Mapping the electrophysiological and morphological properties of CA 1 pyramidal neurons along the longitudinal hippocampal axis. Hippocampus. 2016;26(3):341–361.

4. Patel J, Fujisawa S, Berényi A, Royer S, Buzsáki G. Traveling theta waves along the entire septotemporal axis of the hippocampus. Neuron. 2012;75(3):410–417.

5. Margrie TW, Schaefer AT. Theta oscillation coupled spike latencies yield computational vigour in a mammalian sensory system. The Journal of physiology. 2003;546(2):363–374.

6. Teeter C, Iyer R, Menon V, Gouwens N, Feng D, Berg J, et al. Generalized leaky integrate-and-fire models classify multiple neuron types. Nature communications. 2018;9(1):709.
