# Supplementary material for: Theta oscillations optimize a speed-precision trade-off in phase coding neurons
Source: PLoS Comput Biol. 2024 Dec 2;20(12):e1012628. doi: 10.1371/journal.pcbi.1012628 (PMC11637358; doi:10.1371/journal.pcbi.1012628)
Supplement: S5 Appendix — Details the numerical integration of Eq 1 used in simulations supporting our theoretical framework. (PDF) [file pcbi.1012628.s005.pdf]

S5 Appendix. Simulations

To generate the figures based on simulated data, we discretized the corresponding parameter space (e.g., frequency–noise space) into a 200×200 grid. At each point on this grid, we simulated neurons according to Equation 1 (main text) and the corresponding parameters. The neuron was initialized at $V = 0$ and at its theoretical phase-locking value, i.e., expected phase, $\phi_0 = \mu_\phi$, to simulate steady-state dynamics. Then, Equation 1 (main text) was integrated by the Euler–Maruyama method for a total time encompassing 2 oscillation cycles, with a dt of 0.1 ms. We recorded the phase of the first spike after the beginning of the second cycle, with the trough acting as the origin. This simulated trial was repeated 10000 times for each of the $M = 10$ levels of I_s to obtain the spike phase distributions, characterized by their means μ_i and variances σ_i^2.

To estimate the mutual information comparable to the analytical approximation (described in S3 Appendix), we generated 10000 random samples from each Gaussian distribution $\mathcal{N}(\mu_i, \sigma_i^2)$. These samples were combined to create a normalized histogram of the Gaussian mixture, discretized into 100 bins. Using this histogram, we calculated the marginal entropy $\mathrm{H}(R)$ of the Gaussian mixture via Shannon's entropy formula:

$$\mathrm{H}(R) = -\sum_{l=1}^{L} p_l \log_2 p_l \, \Delta r_l,$$

where p_l is the estimated probability density in bin l, Δr_l is the bin width, and $L = 100$ is the total number of bins. The conditional entropy $\mathrm{H}(R|S)$ was directly calculated using the known entropy of Gaussian distributions:

$$\mathrm{H}(R|S) = \frac{1}{2M} \sum_{i=1}^{M} \log_2 \left(2\pi e \sigma_i^2\right),$$

with M being the number of Gaussian components, corresponding to the different levels of I_s. Lastly, the mutual information was obtained by:

$$\mathrm{I}(S;R) = \mathrm{H}(R) - \mathrm{H}(R|S).$$

The information rate was then computed exactly as described in S3 Appendix.
