# Supplementary figures and images for: Theta oscillations optimize a speed-precision trade-off in phase coding neurons
Source: PLoS Comput Biol. 2024 Dec 2;20(12):e1012628. doi: 10.1371/journal.pcbi.1012628 (PMC11637358; doi:10.1371/journal.pcbi.1012628)

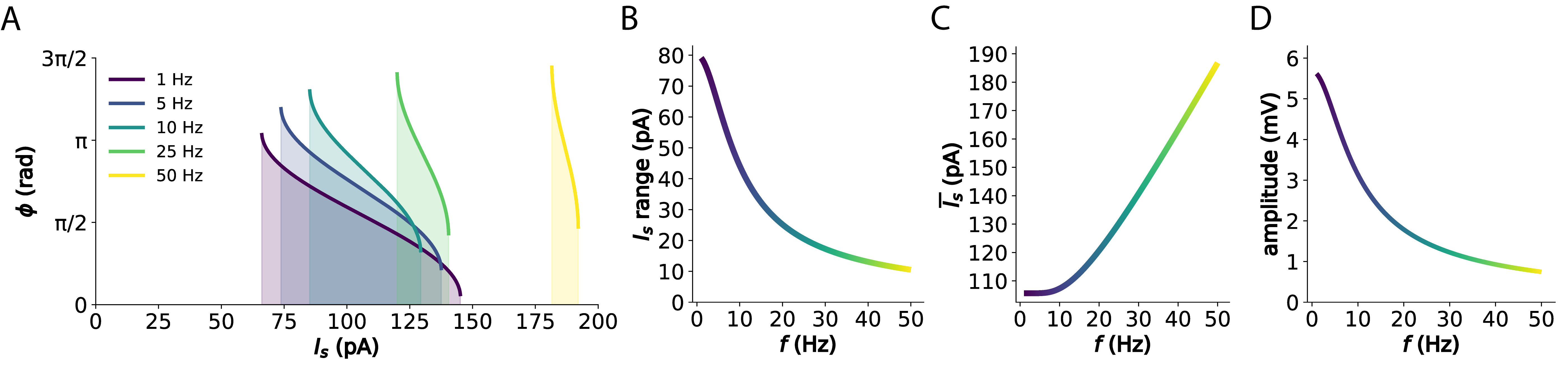

Supplement: S1 Fig — (A) Phase-locking function for different values of the oscillatory frequency f (color coded). Shaded areas denote the phase-locking range of Is, corresponding to the domain of Is in Eq 3. The parameters are the same ones used in [6] and in Fig 1, to match hippocampal physiology (described in Table A in Appendix). Note that the phase-locking range spans half of a cycle and not the full cycle as previously thought, in agreement with recent studies [7]. (B) Length of Is range (max(Is) − min(Is) = 2AIosc) across frequencies. (C) Average Is as Is¯=Vth/(Rm(1-e-T/τm)) (middle point in Is range) across frequencies. (D) Effective amplitude of the membrane potential oscillation, Vosc produced by the oscillatory input Iosc. Given that the membrane acts as a low-pass filter, determined by τm, the effective oscillation in the membrane potential can be found to be Vosc = RmIoscA. Thus, since the membrane filters the oscillatory input as A=1/1+(τm2πf)2, the amplitude of the membrane potential Vosc will decrease with f approximately as ∼ 1/f. (TIF) [file pcbi.1012628.s006.tif]

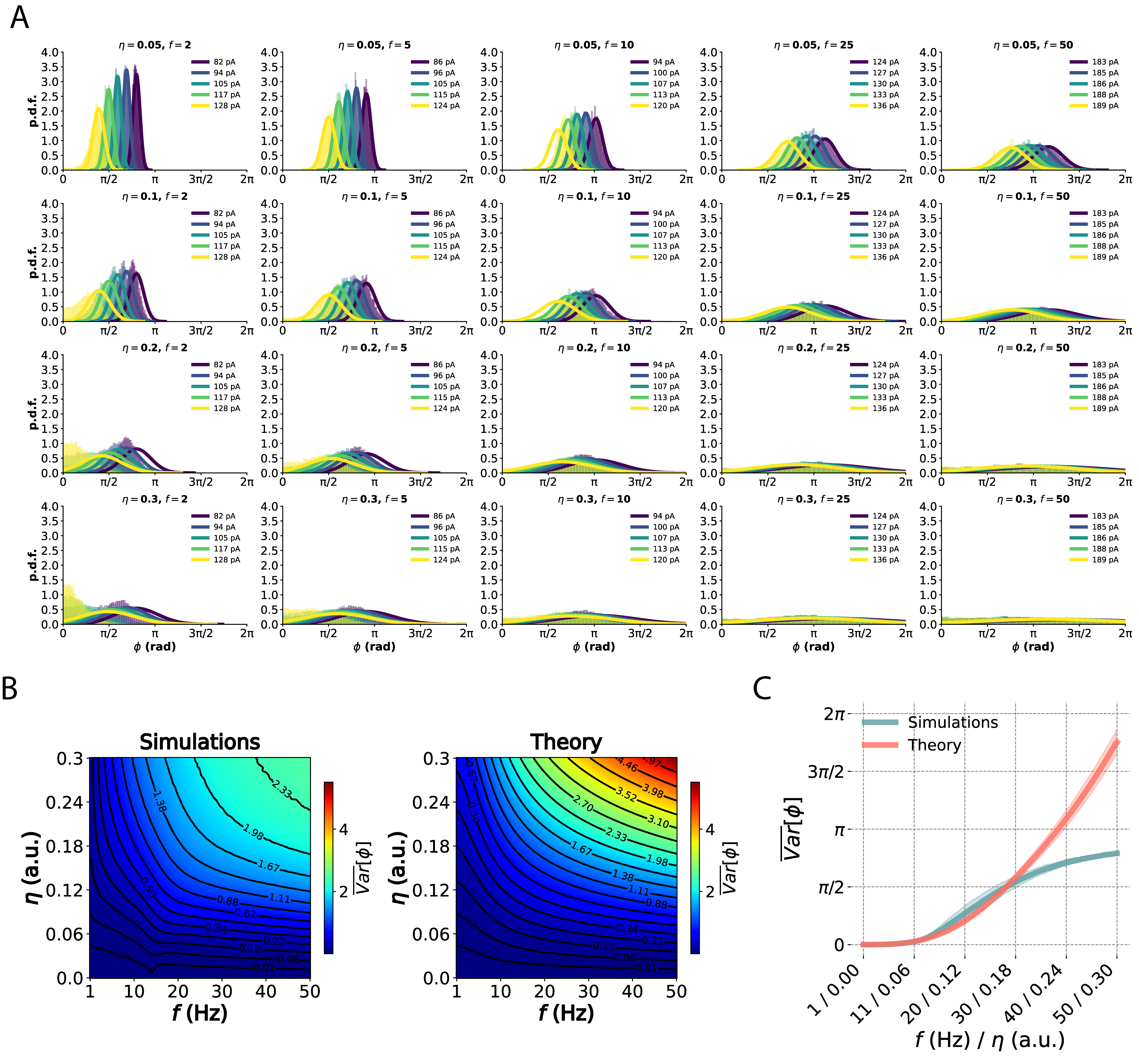

Supplement: S2 Fig — (A) Phase distributions for a range of frequencies and noise strengths. Histograms denote the simulations whereas solid lines denote the theoretical predictions. For the simulations, first-spike phases are recorded from the beginning of the second cycle (with the trough as ϕ = 0), after initializing the neurons to their expected phase ϕ0 = μϕ to allow them to reach steady-state dynamics (as described in Appendix). The parameters used here are described in Table A in Appendix). (B) Average variance in rad2 (across Is levels) across a wide frequency–noise parameter space, for simulations and the theoretical predictions. (C) Diagonal slices of plots in (B), showing the deviation of the theory from the simulations after a certain level of noise amplitudes at high frequencies, due to the bounded variance of simulated spike phases constrained to the measurable range of [0, 2π] radians. (TIF) [file pcbi.1012628.s007.tif]

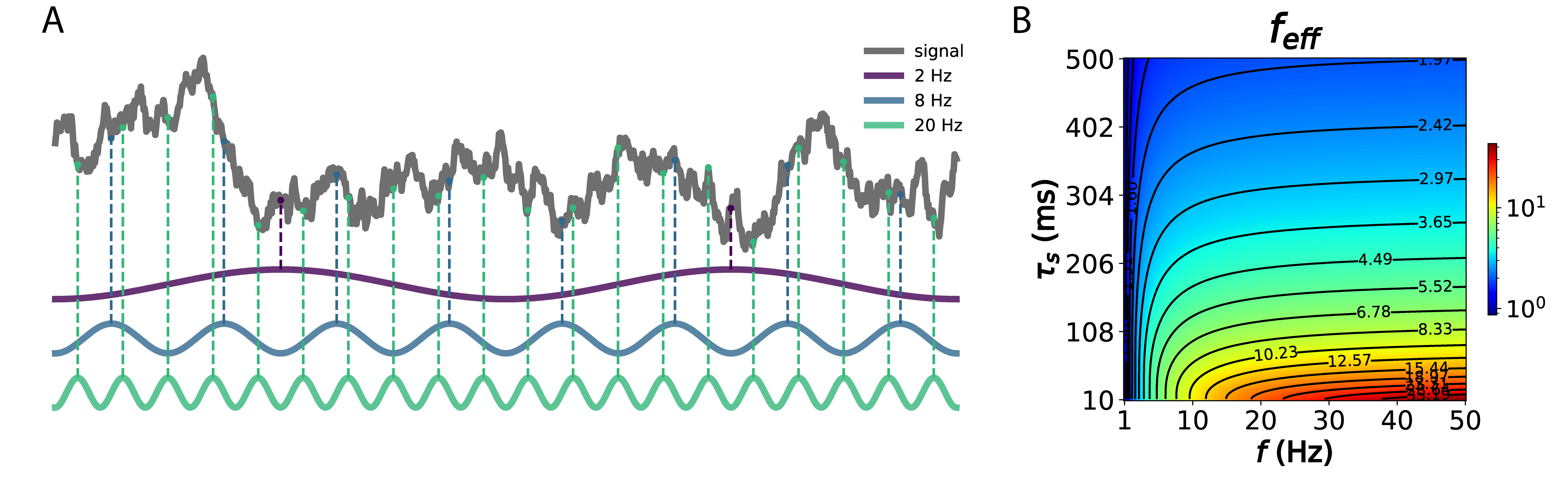

Supplement: S3 Fig — (A) An example signal with τs of 100 ms sampled by different oscillation frequencies. (B) Effective frequency feff=(1-eT/τs)f for various τs values. (TIF) [file pcbi.1012628.s008.tif]

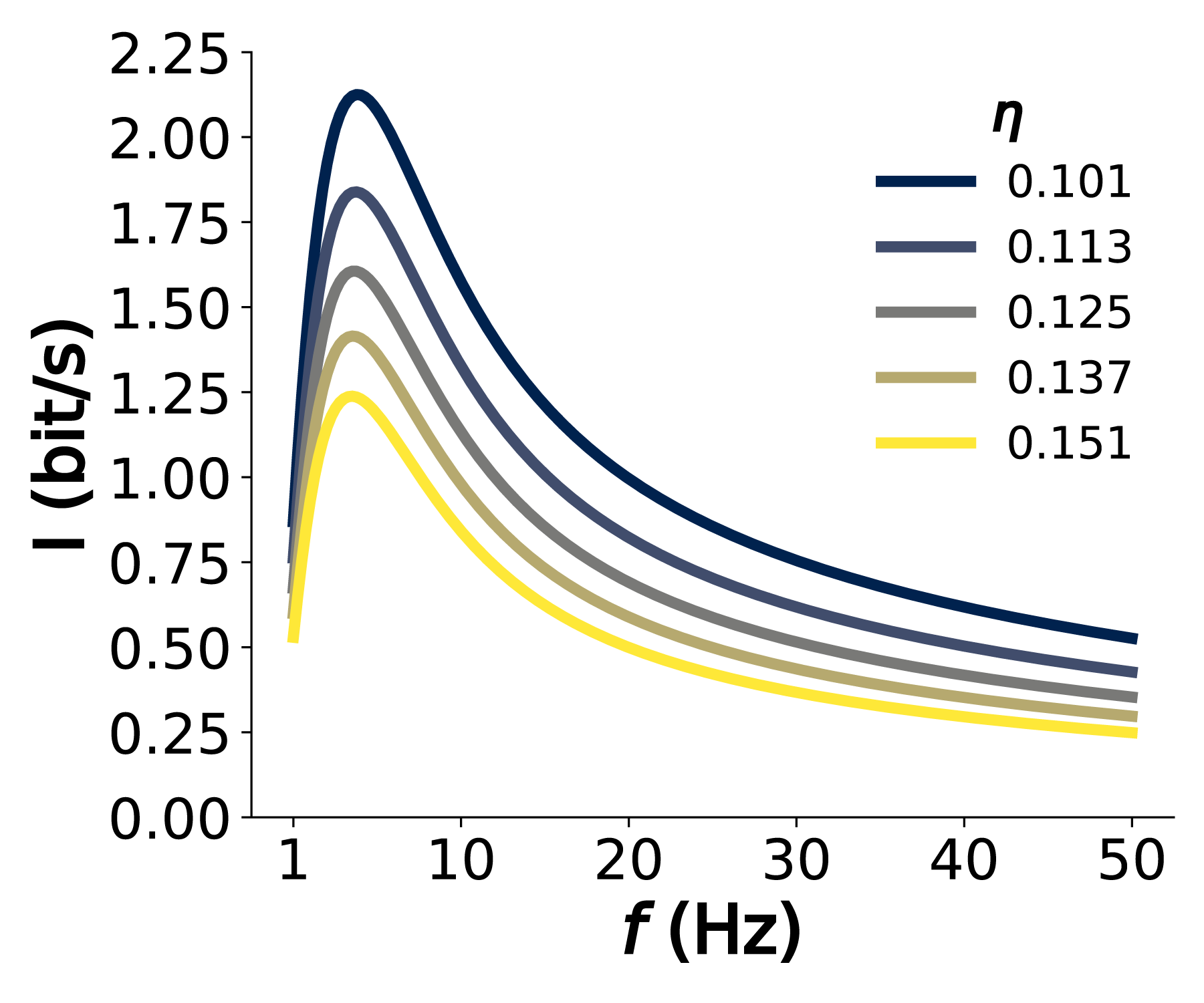

Supplement: S4 Fig — (TIF) [file pcbi.1012628.s009.tif]

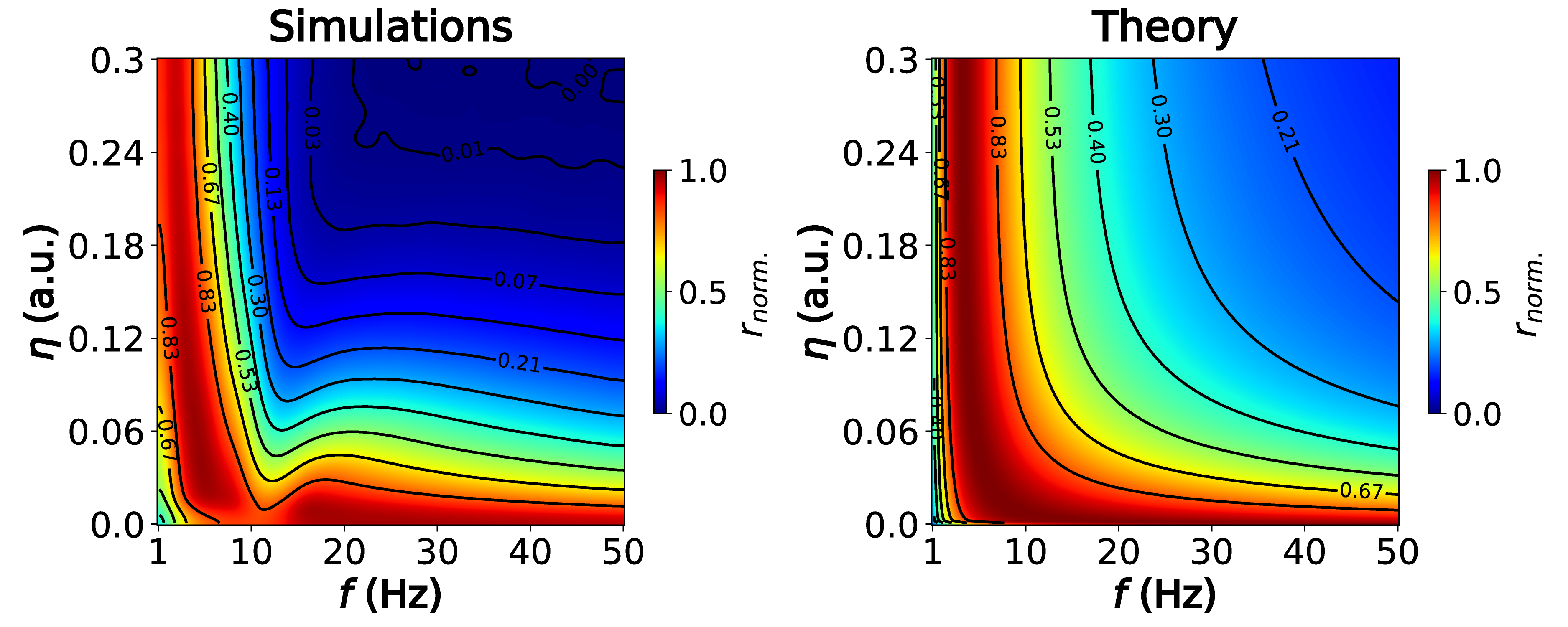

Supplement: S5 Fig — (TIF) [file pcbi.1012628.s010.tif]

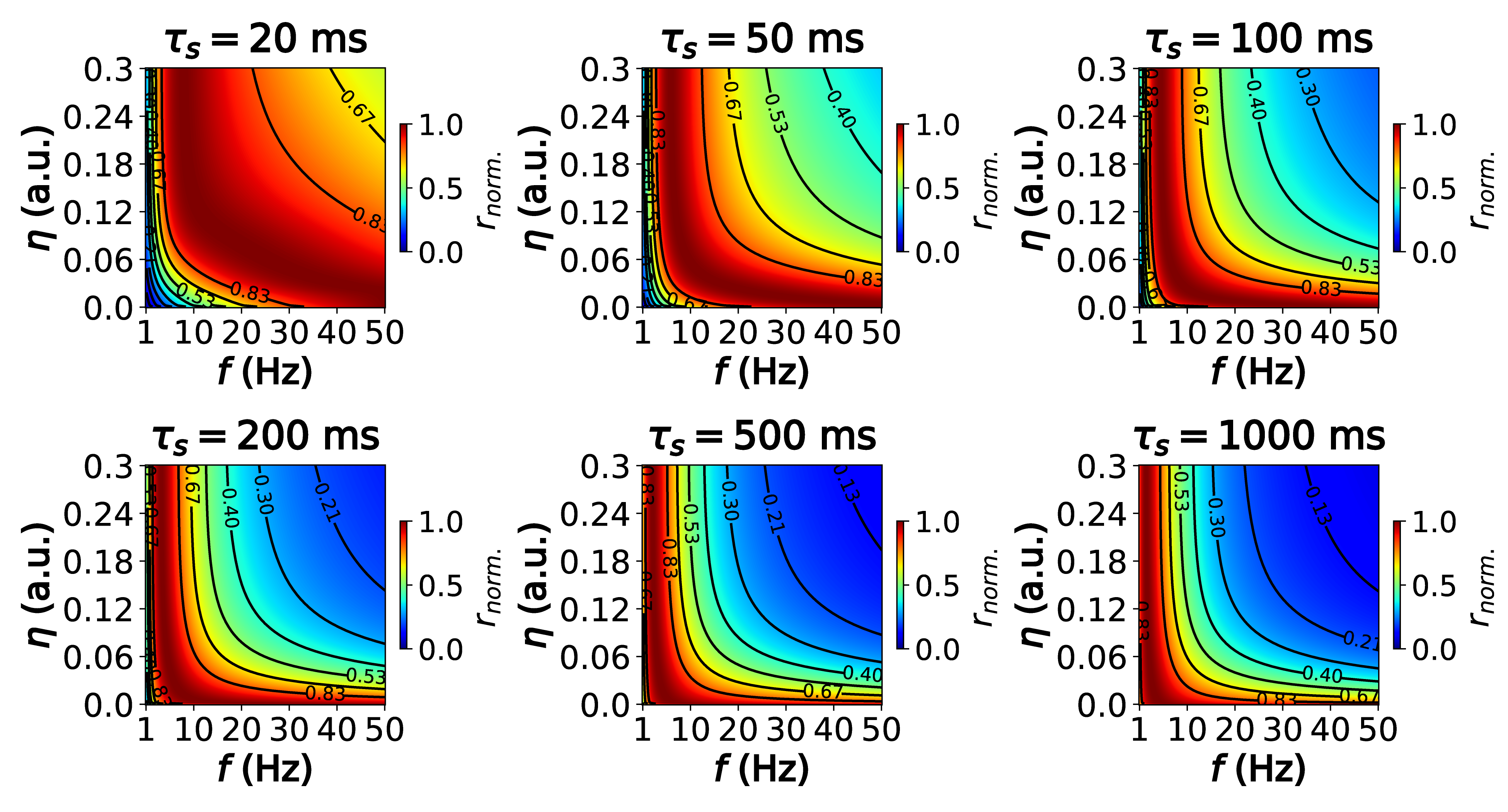

Supplement: S6 Fig — (TIF) [file pcbi.1012628.s011.tif]

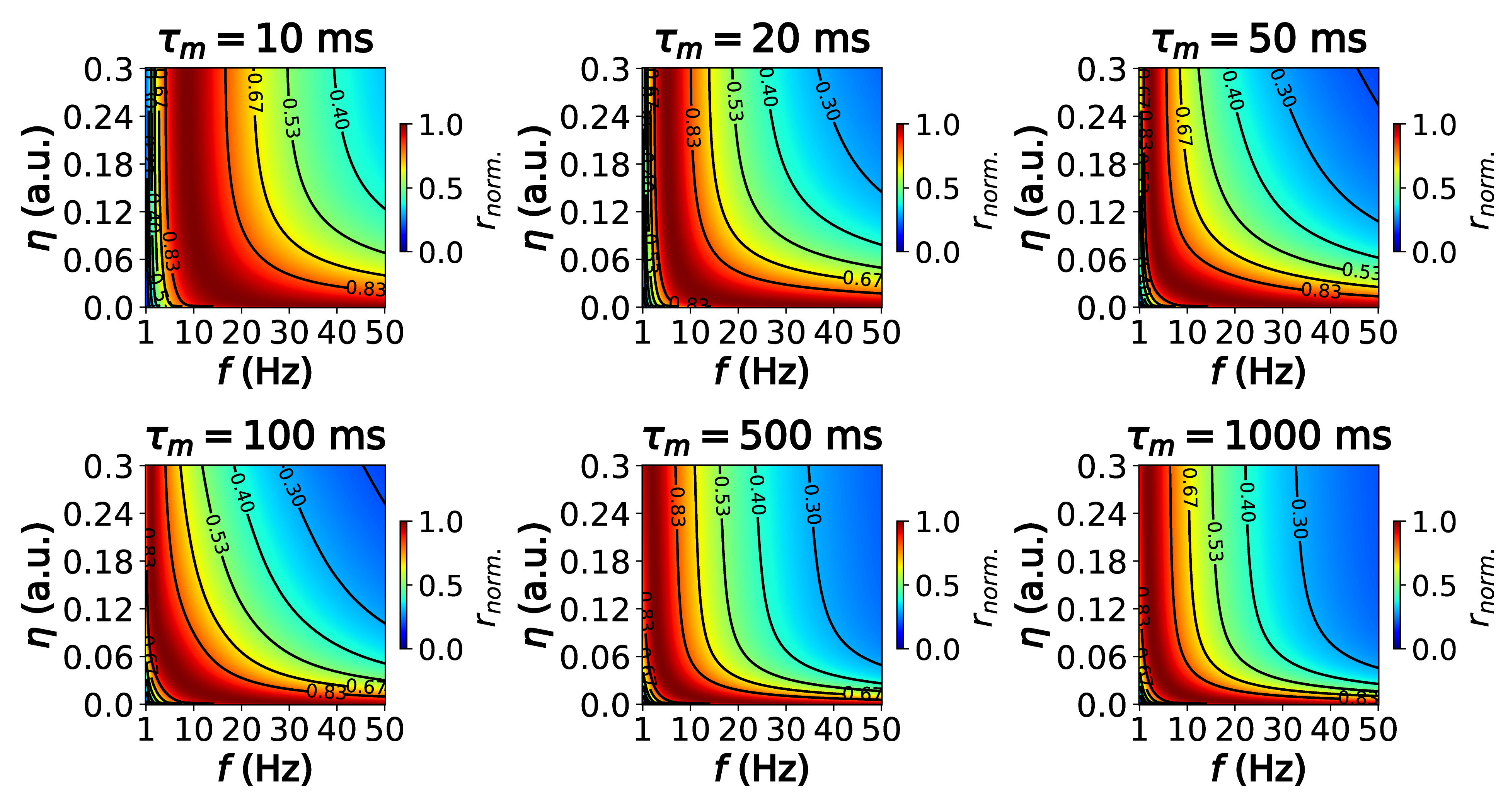

Supplement: S7 Fig — (TIF) [file pcbi.1012628.s012.tif]

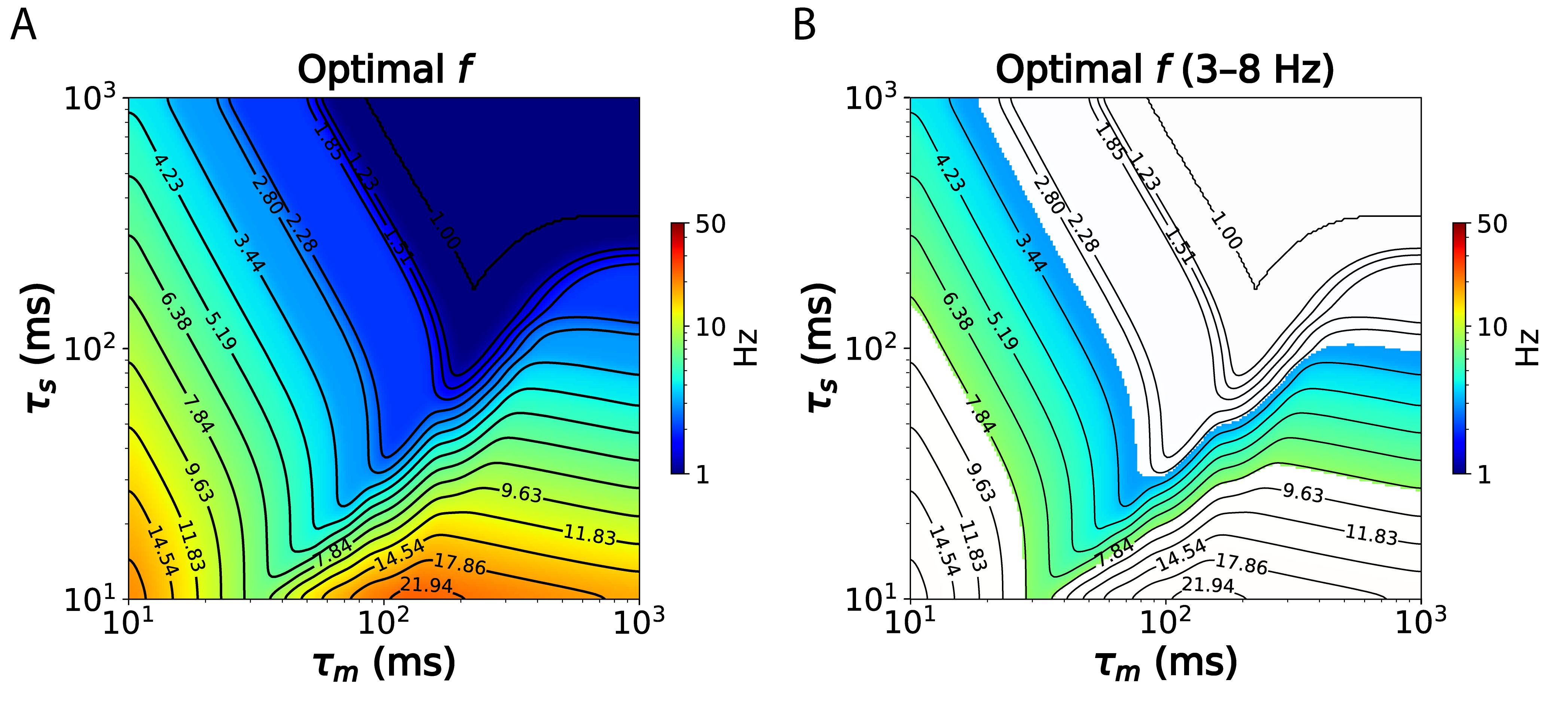

Supplement: S8 Fig — At every point of the τm − τs parameter space (logarithmically discretized in a 200 × 200 grid), we computed rnorm over the frequency–noise space (as in e.g., Fig 4B). Then, the optimal frequency was estimated as an average of the peak frequency between the physiologically-realistic noise range η = [0.1, 0.15]. (TIF) [file pcbi.1012628.s013.tif]

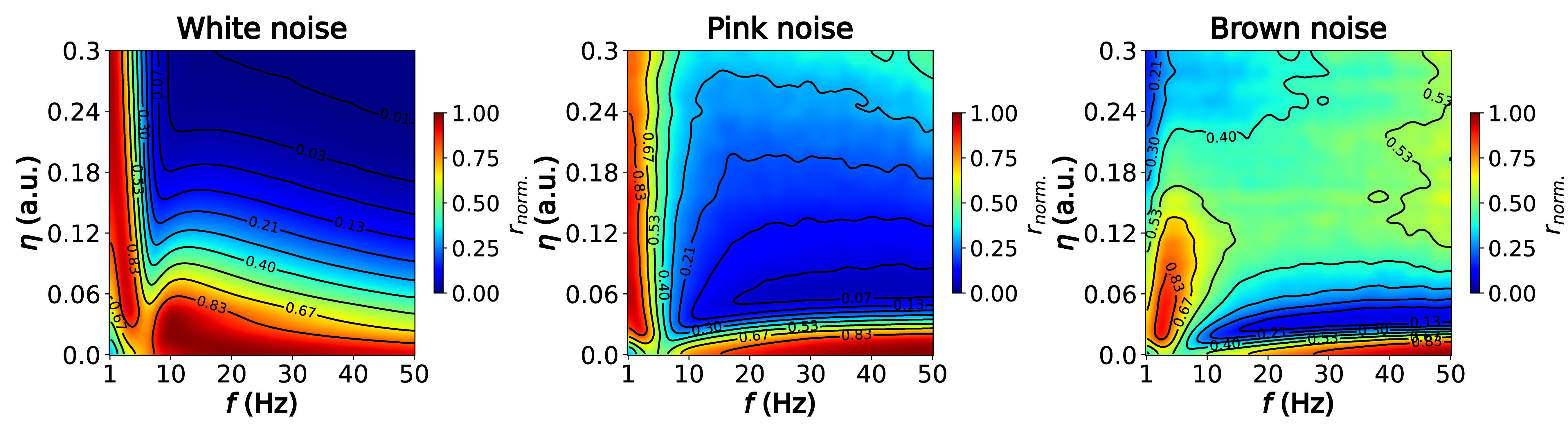

Supplement: S9 Fig — A value of 100 ms was used here for τs. All plots represent the results of simulations. (TIF) [file pcbi.1012628.s014.tif]

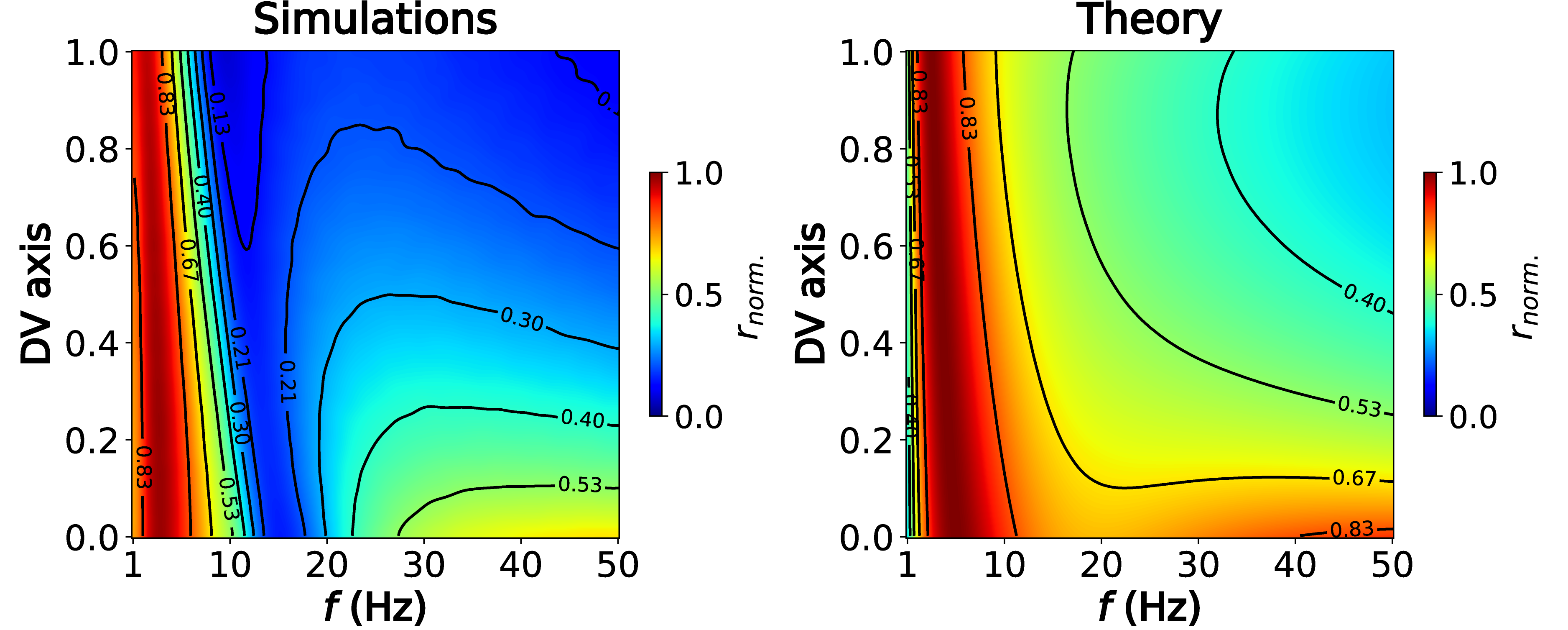

Supplement: S10 Fig — (TIF) [file pcbi.1012628.s015.tif]

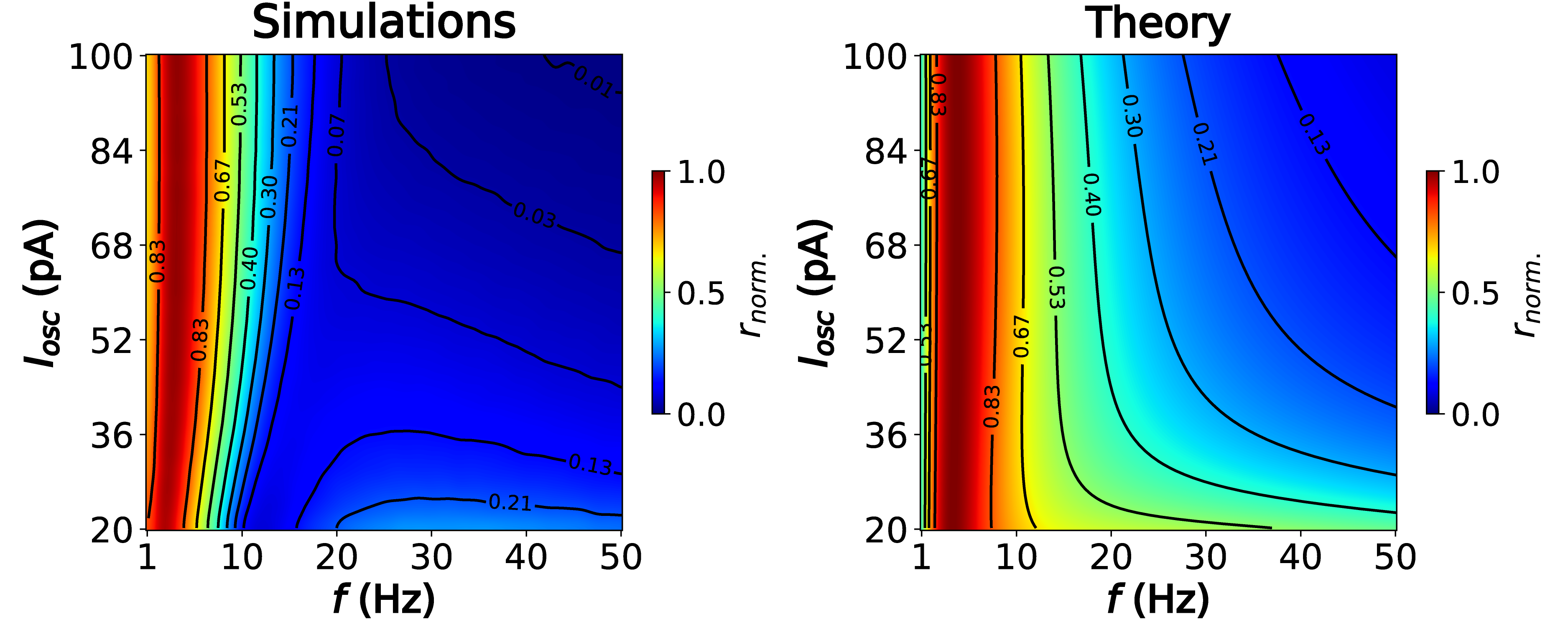

Supplement: S11 Fig — (TIF) [file pcbi.1012628.s016.tif]
