# Supplementary material for: Theta oscillations optimize a speed-precision trade-off in phase coding neurons
Source: PLoS Comput Biol. 2024 Dec 2;20(12):e1012628. doi: 10.1371/journal.pcbi.1012628 (PMC11637358; doi:10.1371/journal.pcbi.1012628)
Supplement: Attachment — Submitted filename: PhaseCoding_ReviewResp_PlosCompBio.pdf [file pcbi.1012628.s017.pdf]

From: Adrian Fernandez Amil
Heyendaalseweg 135 (Huygens building–HG00.137)
6525 AJ, Nijmegen, The Netherlands
Email: adrianfernandezamil@gmail.com

October 6, 2024

To: Daniel Bush (Academic Editor) and Marieke van Vugt (Section Editor)
PLOS Computational Biology
Public Library of Science UK – Nine Hills Road, Cambridge, CB2 1GE, United Kingdom

Dear Daniel Bush and Marieke van Vugt,

We sincerely appreciate the time and effort that you and the reviewers have invested in reviewing our manuscript "Theta oscillations optimize a speed-precision trade-off in phase coding neurons" (previously entitled "Theta oscillations optimize information rate in the hippocampus"; PCOMPBIOL-D-22-01815). We have carefully considered the comments and suggestions provided by the reviewers and have addressed each point in detail (see sections below). In summary, the major improvements can be listed as follows:

- **Analytical approximation of information rate:** We now provide a novel analytical approximation of the information rate (bits/s) of stochastic leaky integrate-and-fire (LIF) neurons performing phase coding, rather than relying solely on simulations. This required deriving new approximations for the variance in spike phase and the entropy of Gaussian mixtures. Importantly, the simplicity of the resulting mathematical expressions makes them suitable as a foundation for analytical studies of phase coding and for drawing principled conclusions.
- **Biologically realistic noise term:** We have updated the neuron's model equations to account for the synaptic origin of noise (i.e., Poisson processes), making the noise amplitude signal-dependent and thus more biologically realistic.
- **Comprehensive supplementary material:** We have included supplementary material that supports the main text and addresses some of the primary concerns raised by the reviewers. This includes additional simulations using pink noise instead of white noise, performing an extensive parameter search (over two orders of magnitude) on the most critical model parameters (primarily the time constants), and exploring the application of our theory to brain areas beyond the hippocampus (e.g., visual cortex and olfactory bulb).
- **Clarification of assumptions and framework:** We have clarified our assumptions and theoretical framework both conceptually and mathematically. Conceptually, we explicitly combine the efficient coding and oscillations-as-sampling hypotheses to justify our focus on information rate. Mathematically, we make the assumptions about the statistical nature of the encoded signal more explicit, leading to a new equation for estimating information rate.

Overall, we have thoroughly revised the main text, updated the figures and references, and added appendices and supplementary material to clarify and support the methods and results presented. Below, we provide a point-by-point response.

Editors:

"In particular, it is not clear how well the findings presented here would generalise beyond the specific implementation and parameter values used, and therefore what the implication of these results is for a broader understanding of the theta rhythm and hippocampal function. In addition, as highlighted by the reviewers, the details of the model examined here are not always clearly described or well justified."

- As mentioned in our Summary above, we have conducted an extensive parameter search, applied our theory to areas beyond the hippocampus, and provided analytical expressions that are manageable enough to rigorously study parameter interactions more generally. In addition, we have described the parameters for all figures and results in tables in the appendix, including proper justifications and references.

Reviewer #1:

"Most analytical results are already published in a paper cited by the authors (McLelland, D. and Paulsen, O., 2009). However the authors make no reference to this information being there. This is true for the derivation of the rate-phase transform in Equation (1), well-studied in that reference, but presented in the results section without explicit mention, and for the use of information theory to characterise the phase code, also well-studied in the reference. Although not the authors' aim, they must be more careful not to unwillingly mislead the reader into thinking these are novel results or approaches. I suggest extensive rewriting to make a clearer distinction between what are the already-published frameworks the authors would like to build on and the novel contributions made by the authors. In fact, given that the major novel results are simulation-based, there's no need to present Equation (1) or Figure (1) in the main text, although it can be added for clarity (emphasis must be made that the result is not new)."

- Thank you for bringing this to our attention. We apologize for any confusion this may have caused and acknowledge that we should have properly cited McLelland and Paulsen (2009) in the main text, rather than only in the appendix. We have now explicitly referenced their paper when introducing the equation for the expected phase-of-firing. The full derivation of this equation—which is not included in McLelland and Paulsen (2009)—is provided in the appendix for readers interested in the details. Additionally, we have clarified the novel aspects of the remaining equations to emphasize the new analytical contributions of our work.

<u>Major</u>

"The key result is to obtain a preferred oscillatory frequency given a set of model param-

eters for the LIF neuron. Given the broad distributions of biophysical neuron properties for a specific cell type in a specific area, it is imperative that the authors show how this preferred frequency depends on the choice of model parameters, perhaps in a stereotypical way through an effective combination of parameters, as the authors start to show for the D-V gradient. This will lend major support to the authors' claim and give the reader an idea of how robust the results are and provide an intuition for why this frequency is preferred by the neuron. In addition, this would generalise the result to other species such as bats and humans (of interest to the broader community but that present a different preferred frequency range). To avoid unnecessary confusion, I suggest the authors use realistic values for all their model parameters (not setting Vreset and Vreset to zero, and a realistic value for Vth, although the LIF depends mostly on the difference Vth-Vreset so results shouldn't drastically change)."

- Thank you for the suggestion. We agree with the Reviewer and have accordingly conducted an extensive parameter search on the most critical model parameters, specifically the membrane time constant and the characteristic time constant of the input signal to be encoded. Please refer to the new Figures S6, S7, and S8, where we explore the parameter space of time constants ranging from 10 ms to 1 s. Figure S8 is particularly noteworthy, as it identifies regions of the parameter space where the theta band is not the optimal frequency. Furthermore, we have generalized our results to extra-hippocampal areas where phase coding has been observed—such as the primary visual cortex and the olfactory bulb—demonstrating that our main prediction holds across a variety of meaningful parameter configurations.

"The authors use a LIF model with white noise. The structure of time-correlations in the noise will greatly impact the phase distribution in the neuron model. It is critical that the authors show how the results change for a more biologically realistic stochastic process, for example pink noise (Ornstein–Uhlenbeck process), as a function of different correlation lengths."

- Thank you again for your suggestion. This was indeed a critical point that we had previously overlooked in favor of mathematical tractability. Although we still use white noise for the main results of the paper, we have now included simulations of hippocampal neurons across a wide frequency-noise space using pink noise and brown noise to represent different correlation lengths (see new Figure S9). Importantly, while pink noise leads to qualitatively similar results as white noise, brown noise—which is unrealistic in neurons—yields different results. We interpret this finding as further emphasizing the specificity of the theta band under biologically realistic conditions.

<u>Minor</u>

"The authors should provide arguments as to why they believe it is a good approximation not to use circular statistics in their analyses, in particular for ranges with high firing rates."

- We appreciate the reviewer's concern regarding the use of circular statistics. In our analysis, we chose not to use circular statistics because phase coding—particularly in the context of phase precession—implies a linear progression from the end to the

beginning of the phase range. Specifically, a spike occurring at the beginning of the cycle represents a strong stimulus (e.g., peak of asymmetric place field), while a spike at the end represents a weak stimulus (e.g., entry of the place field). Using circular statistics would obscure this important distinction, as it treats the phase as a continuous loop without a defined start or end point. Therefore, employing non-circular statistics is not merely a methodological choice but a conceptual necessity to accurately capture the linear nature of the standard phase coding framework.

"The authors should cite studies showing that the LIF model is a good model to reproduce the phase of the first spike when fitting experimental recordings."

- The leaky integrate-and-fire (LIF) model is widely recognized as an effective approximation of excitatory neuron behavior and dynamics, as demonstrated in studies like Teeter et al. (2018, Nature Communications). More importantly, McLelland and Paulsen (2009) precisely showed that the LIF model accurately reproduces the phase of the first spike when fitting experimental recordings.

"It is confusing to use the same notation R for responses and information rate."

- We appreciate the feedback and have revised the notation to eliminate this confusion. We now consistently use 'R' to denote responses and 'r' for information rate, ensuring clarity and consistency throughout the manuscript.

"The authors must improve the readability of the paper. In particular, the paper is made unreadable by displaying equations and symbols without immediate explanation but rather directing the reader to the methods. The situation is made worse after realising that almost no formula is needed to understand the major contributions of the paper, which are mostly simulation based, and can be well-explained with words. Also true for some figures."

- We thank the reviewer for highlighting this issue. To improve readability, we have revised the main text to provide immediate explanations of all equations and symbols when they are introduced, reducing the need for the reader to refer to the appendix. Moreover, detailed mathematical derivations and supplementary figures have been moved to the appendices for interested readers. This revision should improve the paper's readability, with particular importance in this new version since most of our results are now primarily analytical rather than simulation-based.

"Instead of clipping colormaps please consider plotting a logarithmic version."

- We have updated the relevant figures to use logarithmic scales in the colormaps, which enhances visualization and clarity, particularly in regions with extreme differences in absolute values (see e.g., Figure 3 and S8).

Reviewer #2:

Major

"The choice of model: Every model is an abstraction and its choice is determined by the proposed question. Therefore it is vital to provide a clear justification of the chosen model. The here used model is a strong abstraction of the actual place field activity. It assumes that phase precession is the result of a ramping input, but only if the analysis is constrained to the first occurring spike in each cycle."

- Thank you for emphasizing the importance of providing a clear justification for our chosen model. Our phase coding model is based on the rate-to-phase transform described in McLelland and Paulsen (2009), which is conceptually grounded in the assumption that the first spike of the cycle encodes an asymmetric place field that serves as an excitatory input (Mehta et al., 2002, Nature). Recent studies have provided strong supporting evidence for this model, particularly regarding spontaneous phase precession (Sloin et al., 2024, Science; Zheng et al., 2024, Nature Human Behaviour). We have added these references and expanded our justification in the Introduction to clarify the relevance and validity of our model choice. Furthermore, we wish to clarify that our study does not aim to model the precise place field inputs and their temporal dynamics. Instead, our objective is to provide a more general characterization of the rate-to-phase transformation, studying how neurons can encode arbitrary rate-based inputs through phase coding. We have also tried to clarify this throughout the Introduction.

"Furthermore: Real place cells are phase and rate modulated;"

- It is indeed correct that real place cells exhibit both phase and rate modulation. Our neuron model inherently generates firing rate modulations, especially at low frequencies (1-15 Hz); however, we focused primarily on phase coding in this study and did not analyze the rate modulation in detail. Notably, recent research suggests that rate modulation can be relatively independent of phase modulation, potentially conveying non-redundant information (Zheng et al., 2024, Nature Human Behaviour). We have acknowledged this aspect in the Discussion (see "limitations") and clarified that while our model exhibits rate modulation, the implications of possible phase-rate interactions remain unclear and cannot be currently addressed with our analytical model.

"Phase precession spans a full oscillation cycle which makes the input-phase relationship non-unique;"

- Recent studies indicate that spontaneous phase precession may actually span approximately half a cycle, rather than a full cycle as previously thought (Sloin et al., 2024, Science; McLelland and Paulsen, 2009). This is because the second half of the cycle exhibits a much weaker phase precession slope and appears to be more related to theta sequences originating from CA3, associated with memory retrieval, rather than the encoding of cortical inputs from the entorhinal cortex (EC) (Guardamagna et al., 2023, Cell Reports). Therefore, the input-phase relationship remains relatively unique during the first half of the cycle when phase precession is most

prominent. We have updated the manuscript to reflect these findings and to clarify the scope of our model concerning the current understanding of phase precession (e.g., caption of Figure S1).

"The LIF neuron acts as a lowpass filter which might interfere with their measure of mutual information at higher frequencies;"

- The Reviewer is correct that the low-pass filtering property of the leaky integrate-and-fire (LIF) neuron becomes significant as the oscillation frequency increases. In our revised paper, we explore these effects in detail. Specifically, as the oscillation frequency increases, the amplitude of the effective membrane potential oscillation decreases due to the neuron's filtering characteristics. To ensure the neuron reaches its firing threshold and maintains phase-locking despite the damped oscillations, the input current I_s must be increased (see Figure S1a-c). Since I_s originates from a Poisson process, increasing it also raises the noise amplitude (see Noise paragraph in page 4 for details). Consequently, higher oscillation frequencies lead to broader spike phase distributions, which in turn reduce the information encoded per cycle. Importantly, this is not a limitation of our mutual information estimation method but an intrinsic property of the system that imposes constraints on the encoding capacity of LIF neurons at higher frequencies. We describe this process in detail in the new manuscript.

"Input is implemented as current input instead of a synaptic input (which is justifiable for analytical reasons)."

- We acknowledge this point and have updated our model to better reflect the synaptic origin of the input. Specifically, we now make the noise amplitude dependent on the input by assuming that I_s represents the mean rate of a Poisson synaptic input. This modification allows us to maintain the analytical tractability of the model while incorporating greater biological realism by accounting for signal-dependent noise characteristic of synaptic inputs (see Noise paragraph in page 4, and Equations 1 and 2).

"Mutual information: While mutual information is a popular tool to assess higher order correlations between observables it does not provide a deeper understanding. I am still not sure if the here presented results are a trivial consequence of the membrane's filtering properties beyond the obvious effect of the reduced "window of opportunity" at higher frequencies. The information rate is a good choice but it might strongly depend on the chosen parameters of the model."

- In the context of information theory and neural coding, mutual information is a well-established metric for quantifying the amount of information transmitted by neural responses. While the membrane's low-pass filtering properties do influence the neuron's encoding capabilities at higher frequencies, our analysis shows that mutual information can effectively capture the interplay between these biophysical properties and the neuron's information encoding capacity. The observed effects of the low-pass filtering are not merely trivial consequences but reflect important constraints imposed by the neuron's physiology. We agree that the information rate can depend on the model parameters, which is why we have conducted exten-

sive parameter searches (e.g., Figures S6-9) and derived analytical expressions to understand these dependencies comprehensively.

"Phase code model: As mentioned above, why is the analysis limited to the first spike? In this scenario the correlation between phase and input is expected. But how would the result change if more spikes were included?"

- We focused our analysis on the first spike because this is the standard approach in experimental studies of phase precession, and it forms the basis of how phase coding has been conceptualized in the literature. Including additional spikes would probably make sense when introduce other coding schemes, such as rate coding or inter-spike interval (ISI) coding, which involve different mechanisms and complexities that are beyond the scope of our current work. Our aim was to specifically investigate the information encoded in the phase of the first spike to align with the prevailing experimental and theoretical frameworks.

"Place cell firing does increase as the animal traverses the place field with highest rate in the center of the place field. Further, the here employed ramp excitation covers only the first half of the place field, instead of the typical full cycle. In that case, the input-phase relationship is not unique anymore with the additional difficulty that spike phases are less precise in the second half of the place field."

- As previously stated, recent studies have shown that spontaneous phase precession primarily occurs during the first half of the place field traversal (e.g., Sloin et al., (2024) science). The second half of the cycle exhibits a much weaker phase precession slope and appears to be more related to theta sequences originating from CA3, associated with memory retrieval, rather than the encoding of cortical inputs from the entorhinal cortex (EC) (Guardamagna et al., 2023, Cell Reports).

"LIF is a low pass filter: the LIF neuron acts as a low pass filter for the input current which does not only has an effect on the phase but also on the reliability of the phase. How much of the here shown effect is due to this low pass filtering?"

- This point has also been addressed in detail earlier, where we explained how the low pass filtering properties of the membrane affects the reliability of phase by increasing the noise amplitude due to increased inputs necessary to reach the threshold after damping the input oscillation. Therefore, we acknowledge the importance of the low-pass filtering properties of the LIF neuron in shaping its encoding capacity.

"Would the maximum in the theta band change for different τ_m?"

- Thank you for bringing this important point to the discussion. Indeed, varying τ_m can affect the optimal frequency. In Figure S7, we show that the theta band (or more generally, the low frequency range) remains optimal across a wide range of τ_m values (from 10 ms to 1 s). However, our broader parameter search in Figure S9 reveals that τ_m has a nonlinear effect on the optimal frequency, identifying regions where the specific theta band is not optimal.

"As a comment: it has been shown for LIF neurons that filtering properties change dra-

matically when the input is modulated with pink noise instead of white noise (see e.g. Brunel N, Chance F, Fourcaud N, and Abbott L. Effects of synaptic noise and filtering on the frequency response of spiking neurons. Phys Rev Lett 86: 2186 –2189, 2001.). Would that influence the results?"

- Thank you for bringing this to our attention. We have addressed this by incorporating simulations with different types of input noise. In Figure S9, we demonstrate that using pink noise—characterized by a $1/f$ power spectrum—yields qualitatively similar results to those obtained with white noise. This suggests that our conclusions are robust to the introduction of temporal correlations inherent in pink noise. However, when we use brown noise, which has a $1/f^2$ spectrum and stronger temporal correlations, the results deviate significantly. This indicates that while the neuron's filtering properties and the resultant phase coding are influenced by the type of input noise, our primary findings remain valid under biologically realistic conditions represented by white and pink noise.

"Dorsal-Ventral: Along the DV-axis there is a clear shift towards lower frequencies (Figure 5C). Although the theta band is still favored, the optimal frequency clearly shifts. The neuron model is set up as RC-circuit. Thus the membrane time constant τ_m, the membrane resistance R_m and the capacity C are linked through $\tau_m = R_m C$. Here, the model seems to assume that C is constant along the DV axis. Is that true?"

- In theory, since $C = \tau_m/R_m$, variations in R_m and τ_m along the DV axis would imply changes in the membrane capacitance C. However, the ratio τ_m/R_m remains relatively constant, implying that C does not vary significantly in practice. The constancy of the τ_m/R_m ratio is crucial because, as shown in Figure 4E of McLelland and Paulsen (2009), it preserves the rate-to-phase transfer function across the DV axis. This preservation ensures that the effect of noise on the phase distributions remains consistent, supporting our model's assumptions. Therefore, while C may vary slightly, its impact on our results is minimal due to the relatively constant τ_m/R_m ratio along the DV axis. Moreover, it should be noted that other parameters are varied as well, such as the oscillation amplitude I_{osc} and the spike threshold V_{th}, which could in turn change the optimal frequency. However, imprecisions in some of these parameter gradients taken from physiological studies could result in an unbalanced covariation that could lead to the observed slight shift towards lower frequencies within theta along the DV axis. Therefore, we argue that the actual importance of this slight shift in frequency remains unclear.

"As an added effect, the increase of the membrane time constant changes the filtering properties of the neuron towards lower frequencies. Could that contribute to the shift towards lower frequencies in Figure 5C?"

- In our analysis, we found that changes in τ_m alone have nonlinear effects on the optimal frequency, as depicted in Figures S7 and S8. Along the DV axis, τ_m increases from approximately 15 ms to 30 ms. According to our findings in Figure S8, this increase would naturally shift the optimal frequency toward lower values. However, this effect is partially mitigated by a concurrent decrease in membrane resistance R_m, which helps maintain a relatively stable τ_m/R_m ratio. This compensation lessens the shift, keeping the optimal frequency within the theta band. However,

as noted above, we are unsure about the actual implications of such a slight shift, given the concurrent covariation of 4 different parameters in our analysis.

<u>Minor</u>

"Figures 1, 2 and 4 are misleading: Figures 1 A suggests that spikes happen on the ascending as well as descending phase (which is (mostly) not the case here)."

- We have substantially revised the figures. However, we want to note that spikes can also occur on the descending phase when input is weak and the noise amplitude is sufficiently high—a scenario we explore by testing a range of noise levels, including extreme cases. The updated figures now accurately reflect these dynamics (e.g., Figure S2a).

"Figure 2 A shows more than one spike per cycle (here only the first spike in every cycle is considered)."

- While our analysis focuses on the first spike of each cycle for phase coding, we acknowledge that model simulations can produce additional spikes per cycle, especially under strong input conditions at low oscillation frequencies. These extra spikes generally do not impact the phase coding mechanism. However, we also identified very specific cases where there can be cycle-to-cycle interference between extra spikes and the first spike of the next cycle (e.g., see and Figure S2 at f=10 Hz for strong inputs). We discuss the implications of this effect in "limitations" in the Discussion section. Moreover, the theoretical model does not account for them, given the assumptions underlying the derivation of the mean phase-of-firing (McLelland and Paulsen, 2009).

"Figure 4 A again shows a distribution of spike times on the descending phase."

- This point has already been addressed above, where we explain that spikes on the descending phase are possible for weak inputs and high noise amplitudes.

"Figure 3: In 3A the maximum of the phase distribution for low input, low noise and high frequency seems to be on de descending phase of the oscillation (closer to 3 pi/2 than pi). Is this phase shift a result of the filtering?"

- There is a general shift towards later phases with frequency (see Figure S1a) that can be described solely from the rate-to-phase transfer function introduced in McLelland and Paulsen (2009), which we use as the mean phase-of-firing. Indeed, the dominant terms here are "A" and "varphi" in our equation 3 (page 4), which represent the filtering of the membrane and the phase shift controlled by τ_m, respectively. The result of increasing the frequency f (implicit in ω) given the combination of these two terms leads to a positive phase shift toward the descending phase.

"Also, the tick marks on the x-axis along the columns seem to be miss matched."

- Thank you for bringing this to our attention. We have corrected the mismatched tick marks on the x-axis in the figure, which has now been moved to Figure S2a.

Reviewer #3:

Major

"1) "Physiologically constrained" and 'prototypical model. . . ' (lines 247, 294) are used and as the authors mention in their summary, 'how properties of single neurons determine optimal sampling frequencies. . . ', is an outcome of their work. However, it is not always clear what model parameter values are used in different parts of the paper and associated rationale. For example, are the values in Table 1 meant to represent what is used everywhere except when the dorso-ventral aspects are being done? For the speed aspect of the paper, what values are used there? R_m seems out of the range of Fig 5A plotted values, and V_{th} is at end region of Ventral. . . etc. It should be clear and consistent about what model values are used (and why), and providing actual experimental references for the choices should be done, given the 'physiological constraining' that is referred to as a main aspect of their paper. Otherwise, it would give one pause regarding different cellular properties determining optimal sampling frequencies and feels like an over-sell (I think it is really only the membrane time constant that one can physiologically speak to with the models being used?). It would be ok to extrapolate the idea but it should be clear (as noted above)."

- We have addressed this concern by adding detailed tables in Appendix 5.4 that specify the exact parameter values employed for each of the results and figures presented. We have also provided rationales for our parameter choices, supported by citations of experimental studies that justify these values. While the membrane time constant is indeed a critical parameter, other cellular properties are considered (e.g., spike threshold V_{th}), which also shape the neuron's encoding capabilities.

"2) It would be helpful for the authors to expand on the interpretation of 'physiological noise levels' as taken from the cited paper and a particular value (Lansky et al), as this is an important aspect of their work in making their 'theta optimal' take home message."

- We appreciate the suggestion. In response, we have expanded our discussion of physiological noise levels in the manuscript. Specifically, we have added a dedicated paragraph titled "Noise" in the "Neuron model" subsection (page 4), where we detail the noise term, its biological interpretation, and the underlying assumptions. We clarify that we consider a physiologically realistic range of noise levels, encompassing the entire distribution of values reported by Lansky et al. (2006), rather than relying on a single value. Lansky et al. (2006) serves as our primary reference because it directly fits experimental data to the parameters of the stochastic leaky integrate-and-fire (LIF) model, which is the foundation of our own model. Furthermore, we have validated this noise range by checking that values within it yield spike phase distributions with variances comparable to those observed in hippocampal CA1 neurons, as reported by McLelland and Paulsen (2009). We hope that these considerations provide enough justification for the use of "physiological noise levels" in the manuscript.

"3) A recent study from the Moser group (Neuron Volume 109, Issue 6, 17 March 2021, Pages 1029-1039.e8 "Frequency of theta rhythm is controlled by acceleration, but not speed, in running rats" show that theta frequency is not speed-dependent. How would this

affect the authors' results?"

- Thank you for bringing this controversial topic to our attention. While Kropff (2021) suggests that theta frequency is controlled by acceleration rather than speed, recent research has challenged this finding. A comprehensive re-analysis by Kennedy et al. (2022, Journal of Neuroscience) provides evidence supporting the traditional view that running speed, not acceleration, is the primary driver of theta frequency and amplitude modulation. We have updated the Results section pertaining to the DV axis to include these observations and references.

"4) In their discussion, the authors refer to different theta's in human studies, but the well- known type 1/2 theta in rodent (Kramis et al 1975 and many other refs) is never mentioned."

- We apologize for this oversight and thank you for highlighting this important aspect. The distinction between Type 1 and Type 2 theta rhythms in rodents is indeed significant and relevant to our study. We have revised the Discussion section to include this aspect and cite Kramis et al. (1975).

Minor

"1) Eqn 1 has undefined parameters (A, V_{th} etc.) If an equation with various parameters appears in the main text, then its parameters within it should also be defined in main text."

- We apologize for the oversight. In the revised manuscript, we have ensured that all parameters appearing in equations within the main text are clearly defined upon their first introduction, while leaving further details to the appendices.

"2) After Eqn 6, several Figure 2's should probably be Figure 3's."

- We have made substantial revisions to our figures and text, including reordering and renumbering them. As a result, this specific issue no longer applies. However, we have carefully reviewed all figure references throughout the manuscript to ensure they accurately correspond to the updated figures.

We hope that the revisions adequately address the concerns raised by the reviewers. Thank you again for the opportunity to improve our manuscript. We look forward to your further comments.

Sincerely,

Adrian Fernandez Amil
